# “*They are nae worrying about it… so …what’s the point in asking?*”: A qualitative study of candidacy for early CKD care in areas of multiple deprivation

**DOI:** 10.1186/s12882-026-05084-9

**Published:** 2026-06-11

**Authors:** Buse Keskindag, Tom Blakeman, Audrey Hughes, Samira Bell, Mike Melvin, Fumiko Nakamaru, Melany Gray, Bhautesh Jani, Mhairi Mackenzie, Nicole Scholes-Robertson, Eilidh Cowan, Paul Cockwell, Simon Sawhney, Magdalena Rzewuska Diaz

**Affiliations:** 1 Institute of Applied Health Sciences, University of Aberdeen, Aberdeen, UK; 2 Middle East Technical University, Northern Cyprus Campus, Güzelyurt, Turkey; 3 Division of Population Health, Primary Care and Health Services Research, University of Manchester, Manchester, UK; 4 NIHR Greater Manchester Patient Safety Research Collaboration, University of Manchester, Manchester, UK; 5 Patient and Public Involvement Contributor, Aberdeen, UK; 6 Division of Population Health and Genomics, University of Dundee, Dundee, UK; 7 NHS Tayside, Dundee, UK; 8 Aberdeen Council of Voluntary Organisations, Aberdeen, UK; 9 School of Health and Wellbeing, University of Glasgow, Glasgow, UK; 10 NHS Greater Glasgow and Clyde, Glasgow, UK; 11 School of Social and Political Sciences, University of Glasgow, Glasgow, UK; 12 School of Public Health, University of Sydney, Sydney, Australia; 13 Health Determinants Research Collaboration, Gateshead Council, Gateshead, UK; 14 University Hospitals Birmingham NHS Foundation Trust, Birmingham, UK; 15 NHS Grampian, Aberdeen, UK

**Keywords:** Chronic kidney disease, Primary care, Health inequalities, Prevention, Candidacy, Social determinants, Qualitative research

## Abstract

**Background:**

Chronic kidney disease (CKD) is common, associated with substantial comorbidity and strong socioeconomic gradients. Early CKD care focuses on preventing disease progression and reducing cardiovascular risk. However, inequities in access to CKD care are most pronounced early in the disease course, a critical window for equitable population-level prevention within primary care. Understanding how these inequities arise within primary care, where early identification and management largely occur, is therefore essential. This study explored how people living with CKD in deprived areas of Scotland, a setting characterised by marked health inequalities, establish candidacy for early kidney care at a point when prevention should begin but is often lacking.

**Methods:**

A qualitative study informed by critical realism combined semi-structured interviews with 38 adults with CKD (predominantly Stage 3 and primarily managed in primary care) and focus groups or interviews with 23 primary care professionals (*N* = 61). Participants were drawn from the most deprived 40% of neighbourhoods across three Scottish health boards, ensuring variation in CKD stage, diagnosis timing, professional role, and urban-rural setting. Data were analysed using reflexive thematic analysis informed by the Candidacy Framework.

**Results:**

Early kidney care for working-age adults living in deprived areas is often delayed, with CKD detected when personal resources, including time, finances, and support, are already stretched. Diagnosis is typically explained briefly, if at all, leading to uncertainty about its significance and next steps. Communication and care vary widely, with many patients relying on personal persistence to navigate fragmented healthcare systems. Care frequently depends on professional discretion, with uneven community and voluntary resources bridging gaps in care. These patterns reflect structural constraints rather than individual disengagement.

**Conclusion:**

Early kidney care in communities experiencing deprivation represents a constrained preventive window within primary care. The “triple invisibility” of early CKD, at population, system, and symptom levels, undermines sustained engagement at the point when intervention is most effective. Strengthening equitable prevention requires service-level redesign and workforce models that embed kidney health within routine cardiometabolic care, address structural constraints in deprived areas, and integrate physical and psychosocial risk management.

**Trial registration:**

Not applicable.

**Supplementary Information:**

The online version contains supplementary material available at 10.1186/s12882-026-05084-9.

**Table:** 

Contributions to the Literature	
• Provides qualitative insight into how working-age adults and primary care professionals in deprived areas experience and negotiate early CKD care. • Demonstrates how primary care capacity constraints shape inequitable access to progression-preventing interventions. • Shows how socioeconomic precarity interacts with workforce pressures to limit preventive continuity. • Identifies “triple invisibility” (population-, system-, and symptom-level) as a mechanism weakening sustained engagement. • Offers direction for strengthening equitable prevention within universal primary care systems.	

## Background

Inequities in health and healthcare are a major challenge in Scotland (United Kingdom; UK), which has among the lowest life expectancy in Western Europe and a widening gap between the most and least deprived areas over the last decade [22]. Within universal healthcare systems, primary care is the principal platform for population-level prevention. Increasing workload, workforce shortages and rising clinical complexity place strain on the National Health Service’s (NHS’s) capacity to deliver preventive care, particularly for people with complex health and care needs [4, 17, 25]. Recent Scottish reforms aim to strengthen multidisciplinary support and data systems in primary care [1, 44, 45], yet these ambitions remain difficult to realise in routine practice. In high-workload settings, performance metrics that prioritise measurable targets over continuity and communication may further disadvantage deprived populations [41]. In such contexts, chronic illness care strategies can inadvertently reinforce inequalities [32].

Chronic kidney disease (CKD) affects around one in ten adults in the UK [28], with substantial morbidity [18, 50], and is projected to become the fifth leading cause of death globally by 2050 [19, 24]. The international KDIGO classification system explicitly stratifies staging of CKD according to risks of all-cause mortality, cardiovascular events, and kidney-specific outcomes, including progression to kidney failure and acute kidney injury (AKI) [27]. CKD staging integrates underlying cause with assessment of albuminuria and estimated glomerular filtration rate (eGFR), with monitoring and management guided by combined risk category (shown in supplementary material 1). CKD is typically managed within multimorbidity care, including hypertension and diabetes [34], with early detection and management focused on slowing disease progression and reducing cardiovascular risk in line with KDIGO guidance [27].

Reduced kidney function (eGFR) or albuminuria may be present without symptoms and are usually detected only through blood or urine testing [27]. Because the UK does not screen the general population, detection depends on risk-based or opportunistic monitoring in primary care, yet awareness remains low even where testing occurs [18]. National guidance recommends regular kidney checks for people with diabetes, hypertension or cardiovascular disease, with abnormal results confirmed, staged and investigated [37]. In practice, responsibilities vary across teams, and early abnormalities are not always clearly communicated or recorded, contributing to under-recognition [14]. Nephrologists and CKD nurses typically become involved only when progression risk is high, including significant albuminuria, suspected intrinsic renal disease, severely reduced kidney function (stage 4) or established kidney failure (stage 5) [37]. Earlier stages, including mild to moderate kidney impairment, are usually managed entirely in primary care. Although effective risk-stratified approaches to CKD management, including pharmacological and non-pharmacological interventions, are well established [9, 20, 23], their timely use depends not only on detection but on interpretation and clinical action. Gaps in detection, communication, and clinical action shape who becomes aware of CKD early, and who does not, thereby reproducing inequalities in access to early kidney care, defined here as the identification, communication, and management of CKD in its earlier stages.

Evidence from Scotland shows that people in deprived areas experience higher burdens of multimorbidity, later CKD presentation (first identified at a stage requiring referral) and poorer outcomes, including earlier death [12, 42, 43], reflecting structural limits on time, continuity and preventive engagement within primary care. Financial insecurity, limited transport and housing precarity further restrict illness recognition and the capacity to seek and sustain care [43]. Yet little is known about how these conditions shape people’s entry into early kidney care and the interactions through which access is negotiated. Understanding how people’s circumstances shape their ability to seek and sustain kidney health is essential for designing interventions that reduce inequalities. The Action Plan for Scotland commits to reducing social and geographic inequalities in CKD through earlier detection and equitable monitoring [29], but realising these aims requires understanding how candidacy for early care unfolds within constraints of time, continuity, trust and broader structural determinants [36]. Although treatment burden and organisational barriers have been described for older people with advanced CKD [26, 40], little is known about how working-age adults living in deprived areas experience early kidney care.

One framework that helps explain how access is shaped by social and organisational conditions is the Candidacy Framework [16]. It has been applied in studies of chronic and clinically mediated conditions where symptom ambiguity and structural disadvantage influence how people come to be recognised - and to recognise themselves - as eligible for care [30, 33, 46]. Early CKD care shares these characteristics: identification is typically incidental and clinically mediated rather than symptom-led, and opportunities for early engagement are constrained by socioeconomic and organisational pressures [14]. The Candidacy Framework has therefore been used in this study to understand how early kidney care is accessed and navigated from the perspectives of working-age adults living in areas of multiple deprivation (i.e., disadvantage across income, employment and service access domains) in Scotland and the primary care professionals who support them.

## Methods

### Study design

This qualitative study, conducted between July 2024 and May 2025, explored experiences of early kidney care among working-age adults living with CKD and the primary-care professionals who support them in socioeconomically deprived areas of Scotland. Reporting followed the COREQ checklist [49] and was informed by a critical realist orientation recognising the interplay of structure and agency [21]. The study was co-designed and conducted in partnership with patients, public contributors, and professionals, and their contributions are reported following GRIPP2 Short Form guidance [47]; full details are provided in supplementary material 2.

### Study sites

Participants were recruited across three Scottish health boards - NHS Grampian (north-east), NHS Fife (east), and NHS Greater Glasgow and Clyde (west) - selected to reflect geographical and socioeconomic variation in CKD service provision. Additionally, a Scotland-wide community/self-referral route was available, enabling eligible patients from beyond the three participating health boards to take part, which resulted in one participant from outside the three boards.

Participant recruitment continued until information power was achieved to meet analytic depth [35]. Information power was considered across the study aim, sample specificity, quality of dialogue, theoretical engagement, and the analytic strategy employed, informing judgments about sample adequacy rather than relying on numerical thresholds [35]. A study is considered to have adequate information power when the sample is well aligned with the study aim, the participants have relevant experience, the data are of high quality, and the analytic approach allows for in-depth, reflexive interpretation.

### Patients’ eligibility and recruitment

Eligible patients were aged 18–65, aware of a chronic kidney disease (CKD) diagnosis or a sustained acute kidney injury (AKI) requiring ongoing kidney monitoring, confirmed by their GP records, and living within the most deprived 40% of neighbourhoods in Scotland (Scottish Index of Multiple Deprivation [SIMD] quintiles 1–2). Awareness of diagnosis was required to ensure meaningful engagement and to avoid inadvertent disclosure [14].

Eligible patients were identified through medical record screening using routinely recorded diagnostic codes, age and postcode, and were invited via NHS Research Scotland (NRS) Primary Care Networks, nephrology clinics and community organisations. Most NHS participants were identified through primary care records. Recruitment was based on the most recent recorded CKD stage (or self-reported where records were unavailable); where NHS consent was provided, medical record extracts confirmed CKD stage and most recent eGFR. Of 297 study invitations issued by post across the three boards (106 Greater Glasgow & Clyde, 56 Fife, 135 Grampian), 28 primary care patients participated. In secondary care, nephrology staff invited eligible patients during scheduled appointments; of 14 who expressed interest, nine took part. Community outreach through Kidney Research UK, the Grampian Kidney Patient Association and the Scottish Kidney Federation yielded 10 responses, with one meeting eligibility criteria and subsequently recruited. Approximately 12% of invited individuals participated. Rather than aiming for statistical representativeness, the sample provided contextual and experiential diversity across CKD stages, ages and socioeconomic backgrounds aligned with the study aims.

Thirty-eight individuals (*n* = 38) living with kidney disease in socioeconomically deprived areas participated in the study. Of those with available postcode data, 27% resided in SIMD Quintile 1 and 64% in Quintile 2; an additional ~ 9% had deprivation status verified by the NRS Primary Care Network but not disclosed to the research team. Most participants were recruited from large urban areas, with a smaller number from accessible rural areas and small towns, reflecting the geographic distribution of the Scottish population. Most participants had a stage 3 CKD diagnosis and, within this group, stage 3a (mild to moderate reduction in kidney function) was more common than stage 3b. Smaller numbers were at earlier stages of mild kidney function reduction or at more advanced stages of kidney function decline, and one participant had sustained an AKI. Nearly all participants reported multiple physical health comorbidities. Detailed patient participant characteristics, including demographic, socioeconomic, and clinical information, are presented in supplementary material 3 (Table S3.1).

### Professionals’ eligibility and recruitment

Eligible professionals were general practitioners, practice nurses, pharmacists, and reception/administrative staff working in practices serving socioeconomically deprived populations, including “Deep End” general practices (surgeries serving Scotland’s most disadvantaged communities). Professionals were invited via digital flyers distributed through NRS coordinators and practice networks. In total, 23 professionals participated (from 27 who expressed interest): 15 GPs, six practice nurses and two community pharmacists, spanning urban and rural contexts. Detailed professional participant characteristics, including role, data collection method, and health board, are presented in supplementary material 3 (Table S3.2).

### Data collection

Semi-structured interviews and focus groups were conducted by trained and experienced qualitative researchers, primarily by BK, with two patient interviews conducted by MRD, using topic guides co-designed with patient and public partners, piloted and refined iteratively (supplementary material 4). Patients took part in one-to-one, semi-structured interviews conducted by telephone (*n* = 30), online (*n* = 4), or in person on university premises (*n* = 4), typically lasting 41–72 min. Professionals participated in online focus groups (four groups of three to four participants, ~ 90 min) or, where scheduling was difficult, one-to-one interviews (~ 60 min; *n* = 9). Participants were offered interpreter support; one patient interview was conducted in a language other than English at the participant’s request by a bilingual interviewer. No additional non-English interviews were requested. Interviewers had no prior clinical relationship with participants. Participants were informed of the researchers’ roles, study aims, and interest in understanding access to early kidney care in areas experiencing deprivation. All sessions were audio-recorded, professionally transcribed, anonymised, and accuracy-checked. Structured field notes captured contextual details, cross-site differences, and reflexive observations informing ongoing analysis. No repeat interviews were conducted.

At the start of each patient interview, a brief questionnaire captured demographics (e.g. age, gender, postcode, employment) and kidney status at diagnosis and currently. For NHS-recruited participants, kidney health information was verified from medical records by NRS Primary Care Network coordinators and the recruiting nephrologist (or delegated clinician) and, with participants’ consent, securely shared with the clinical team member or the project researcher. All clinical information shared with the research team was pseudo-anonymised and transferred via secure NHS channels.

To reduce barriers to participation, all participants received easy-read study information and were offered a choice of written, online, or verbal consent covering participation, recording, and use of anonymised quotations. Patients received a £25 shopping voucher, and professionals were reimbursed £97 per hour (directly or via general practices), in line with NIHR payment guidance [38] and ethical arguments for fair recognition under sustained workload pressure [6].

### Researcher reflexivity

The team brought complementary disciplinary and practice perspectives, including counselling psychology, general practice, nephrology, clinical epidemiology, health psychology and implementation research. During fortnightly analytic meetings, researchers presented new data extracts and emerging interpretations, which were discussed collectively with patient and public contributors and clinical team members to challenge assumptions and refine analytic decisions. These cross-disciplinary discussions supported sustained reflexivity, alongside memo-writing and explicit attention to researcher positionality throughout data collection and analysis. Additional one-to-one input from lived-experience contributors further strengthened interpretive rigour. Methodological oversight for use of the Candidacy Framework was provided by a medical sociologist.

### Data analysis

Transcripts were analysed using reflexive thematic analysis (RTA) [7] within a critical realist frame [21]. A critical realism approach recognises that phenomena such as access to care are shaped by underlying social structures, while being known through participants’ lived accounts. It guided attention to both participants’ experiences and the generative mechanisms influencing engagement with kidney care, allowing analysis to account for the interaction between individual circumstances and broader organisational and systemic contexts. RTA proceeded through six iterative phases: (1) familiarisation, (2) systematic inductive coding, (3) theme generation, (4) theme reviewing, (5) defining and naming themes, and (6) reporting [7]. This approach emphasises researcher reflexivity by recognising the active role of the researcher in knowledge production, prioritises analytic depth and interpretive richness over consensus between coders, and involves an iterative process of theme development in which themes are generated, reviewed, and refined through sustained engagement with the data rather than assessed through inter-rater reliability. Themes were developed inductively and subsequently interpreted through the domains of the Candidacy Framework [16], which has been widely applied to theorise access to primary care [46].

The Candidacy Framework conceptualises access to healthcare as a dynamic and negotiated process shaped by interactions between individuals and services, rather than a fixed state of eligibility. Its domains comprise seven overlapping stages representing candidacy for a health service, which are defined and adapted for this study in Table 1. Because early CKD is associated with non-specific symptoms and is often detected incidentally, identification was clinically mediated rather than patient-led, with sense-making occurring after disclosure and shaped by multimorbidity; candidacy domains are therefore reported in an empirically informed order. An additional cross-cutting meta-theme (*precarity shaping the conditions for early kidney care*) was developed, rather than forcing the data fit within the existing domains. Together, the domains, presented in an empirically informed order, and the cross-cutting meta-theme structure the presentation of findings in the Results section. Detailed coding trees for both study subpopulations (patients and primary care professionals) are available via the University of Aberdeen online repository [51]. NVivo (R1 2020) supported organisation; in line with RTA, we prioritised reflexive, dialogic sense-making over inter-rater reliability. Themes were built collaboratively with patient partners, checked across subgroups and negative cases, and documented in analytic memos and debriefs. Sampling sufficiency was assessed using the principle of information power [35], based on the relevance, richness and diversity of data in relation to the study aim.

**Table 1:** Definitions of candidacy domains in this study

Candidacy domain	Definition
Identification and early sense-making of kidney disease	How CKD was detected and initially interpreted as important or negligible within routine multimorbidity care, focusing on how patients and professionals recognise and make sense of kidney disease in everyday practice.
Appearing in services - primary care encounters	How kidney disease was communicated and voiced in clinical encounters, and how interactions shaped whether patients felt recognised, reassured, or dismissed.
Navigating kidney disease-related care and support	The practical work patients undertook to pursue kidney-related follow-up within primary care-led systems after identification - including arranging appointments, attending tests, and seeking practical or social support where needed to make care feasible.
Permeability of primary care healthcare services	How the design and organisation of general primary care services enabled or constrained access to kidney-related care, including appointment systems, administrative interfaces, digital routes, geography, and follow-up practices.
Adjudications of candidacy	How professionals judged, prioritised, and acted on patients’ eligibility or capacity for care in the context of multimorbidity and deprivation.
Offers and resistance to kidney care	How offers of kidney care were made, interpreted, and taken up or deferred, shaped by trust, understanding, perceived relevance, and practical feasibility within constrained contexts.
Operating conditions shaping candidacy in kidney care	How system-level contexts - including workload, resources, incentives, and policy - shaped the consistency, continuity, and responsiveness of kidney care, influencing who was prioritised and supported over time.
Precarity shaping the conditions for early kidney care^¥^	How socioeconomic precarity and health-related vulnerability, shaped by structural conditions, influenced patients’ capacity to engage with kidney care before and after diagnosis.

^¥^ a cross-cutting inductive meta-theme, reflecting experiences that extended beyond the seven candidacy domains

## Results

The sample included 38 patient participants, with equal representation of men and women. Most were aged 50–65 years, with the majority having stage 3 CKD, alongside representation from those at earlier and more advanced stages. In addition, 23 primary care professionals were recruited across NHS health boards, including general practitioners, practice nurses, and community pharmacists, providing multidisciplinary perspectives.

**Fig. 1:**
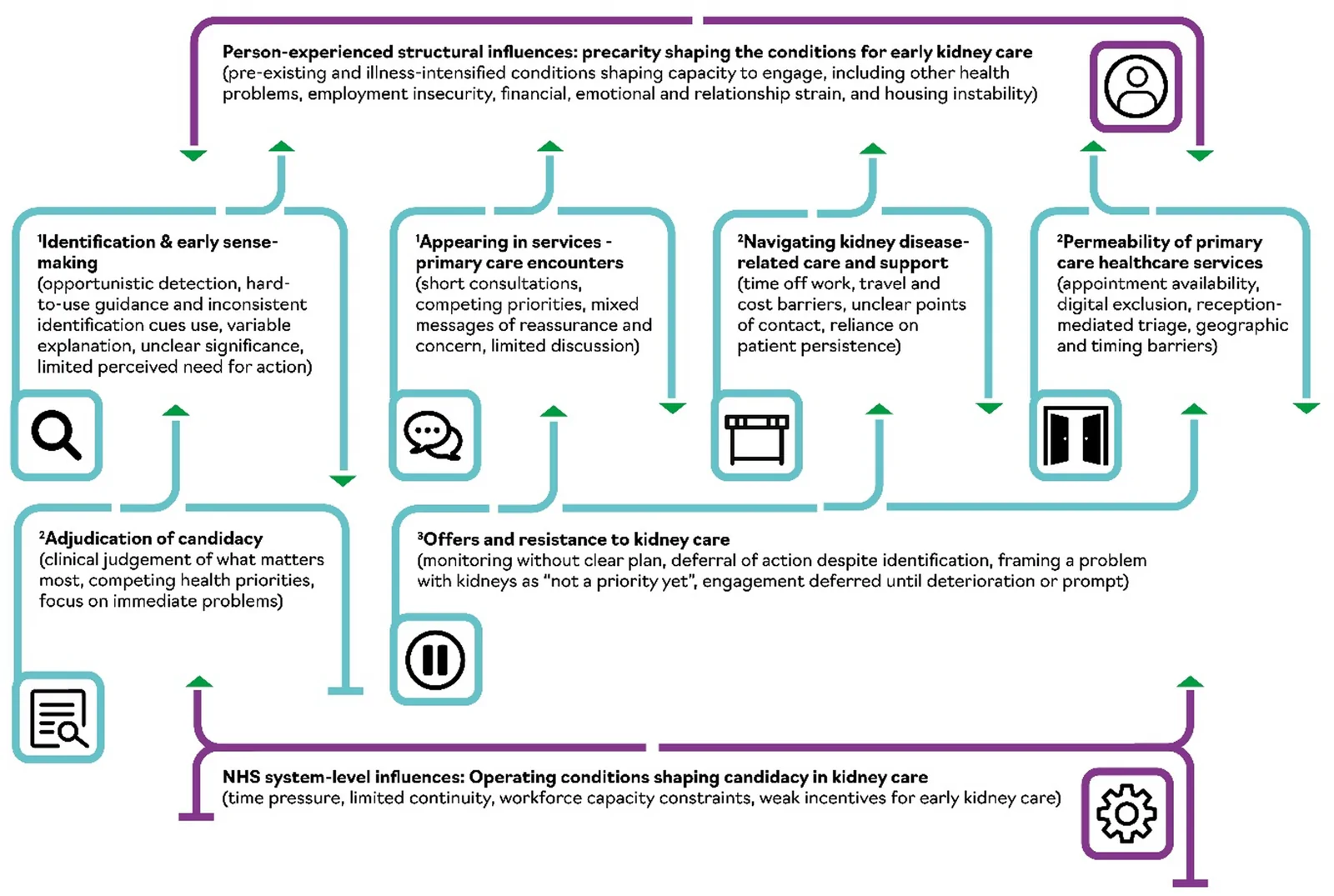
Analytic representation of candidacy for early kidney care in areas of multiple deprivation. The figure illustrates how candidacy for kidney care is negotiated through organisational processes (blue pathways), from identification and sense-making to offers and responses, with non-linear movement that may involve revisiting earlier points. Processes are grouped for interpretive clarity as early¹, mid-process², and later responses³; these groupings are descriptive rather than sequential. Organisational processes are shaped by two cross-cutting influences –primary care operating conditions and structurally shaped socioeconomic precarity and health-related vulnerability experienced by patients – which contextualise candidacy across the pathway rather than constituting discrete steps

The initial sections present findings on how kidney disease was negotiated within primary care systems across the early stages of candidacy, from detection and interpretation through to access, professional decision-making, and responses to care. We begin with identification and early sense-making, as CKD was typically detected incidentally through routine primary care activity rather than through patient-led symptom recognition. We then examine how kidney disease featured within primary care encounters, where its significance was negotiated amid multimorbidity and time-limited consultations, followed by navigation, capturing the work patients undertook to pursue kidney-related follow-up and support. Subsequent sections address the permeability of primary care services, professional adjudications of candidacy - operating across identification and offers of care - and patients’ responses to these offers as organisational processes. We conclude with two domains describing the conditions shaping these processes: operating conditions (NHS system-level influences) and socioeconomic precarity and health-related vulnerability, shaped by structural conditions, that influence patients’ capacity to engage. Together, these contextualise candidacy across the pathway. An analytic representation of these processes is shown in Fig. 1.

### Identification and early sense-making of kidney disease

Primary care professionals described CKD identification as largely opportunistic, emerging through routine blood tests rather than symptoms: “*We get very*,* very few patients presenting acutely unwell with acute kidney injuries*” (GP 4). Detection typically occurred during reviews for other long-term conditions such as diabetes, hypertension, thyroid disorders, or pregnancy complications: “*They were doing blood tests for another condition… and that’s where the kidney function showed as being low*” (Patient 23). For patients, this often meant the diagnosis arrived unexpectedly, with limited understanding of why blood or urine tests had been undertaken or what the findings were intended to assess.

Professionals viewed identification as both a technical and interpretive process. Some followed local renal protocols, while others referred to national guidance such as NICE or SIGN, noting that thresholds and calculators could be “*not very user-friendly*” (GP 7). Most described CKD as best integrated within existing multimorbidity reviews rather than through dedicated clinics: “*You don’t need another whole clinic… you maybe just need to add an ACR onto the bloods you are doing*” (GP 9). Others used practice audits, such as the “triple whammy” medication review (i.e. the concurrent use of a renin-angiotensin-aldosterone system blocker, a diuretic, and a non-steroidal anti-inflammatory drug, which is associated with increased risk of acute kidney injury) [8] to prevent avoidable harm. These accounts positioned identification within routine chronic disease management rather than as a distinct event.

For patients, identification was less about clinical detection and more about making sense of early, often non-specific (attributable to other co-existing health problems) or absent symptoms, resulting in kidney disease being experienced as *invisible*. Many lacked prior knowledge of kidneys or their function: “*I wasn’t very knowledgeable about kidney disease*,* so I didn’t really know what it meant*” (Patient 1). Reactions ranged from shock and disbelief to mild concern or resignation. Some were startled that a chronic disease could be discovered incidentally: “*It was a shock to the system because I had always thought I was fit and healthy*” (Patient 1). Others accepted it with little alarm: “*It does sit at the back of your mind… but it’s not something I dwell on*” (Patient 22).

### Appearing in services - primary care encounters

First conversations about kidney function were often led by practice nurses or pharmacists during reviews for other conditions, positioning them at the frontline of disclosure. CKD diagnosis was a key moment of appearance, marked by uncertainty about what the findings meant and whether further action was needed. Some patients were unsure if they had ever received a formal diagnosis: “*Up to date*,* my GPs etc. have never been concerned I’m having problems with my kidneys… nobody’s really said anything*” (Patient 8). Others only discovered their condition indirectly: “*They told me because I was borderline for kidney disease… that was the first time I knew anything about it*” (Patient 4). In some cases, the information was presented so casually that it did not register as significant: “*When I was told about this*,* it was made very little of… I wouldn’t say I was given that impression at all*” (Patient 23).

This uncertainty was reinforced by how kidney disease was positioned relative to other health priorities. Several patients and professionals described kidney disease as a secondary concern, overshadowed by other chronic conditions such as diabetes or hypertension. One patient participant reflected that “*the kidneys have become a secondary thing… I was quite happy pedalling along*” (Patient 2), while clinicians often prioritised managing comorbidities first: “*If they are coming for their annual hypertension review… they might not be aware of the CKD that they’ve got related to that*” (Pharmacist 1). This framing reinforced uncertainty about the significance of CKD and contributed to patients interpreting silence or reassurance as a sign that nothing more needed to be done.

Against this background, the way results were communicated at this stage determined whether patients viewed themselves as needing further care or assumed nothing more was required. Some recalled being given medication without explanation - “*They just palmed me off with medication*” (Patient 13) - while others described feeling confused and anxious as a result of not knowing what their results meant: “*The kidney count… is quite low… once you hit 50 you hit a bit more serious waters… nobody has discussed any of that with me*” (Patient 23). In time-pressured settings, clinicians tended to soften or abbreviate disclosure - “*The kidneys are just getting a bit slower…*” (GP 4) - while those with greater continuity favoured directness: “*Maybe I’ve made it a bit too reassuring… maybe my letter should actually be saying*,* ‘You really need to look after yourself!*’” (GP 7). These differing approaches influenced how candidacy was voiced and heard, often leaving patients uncertain about whether they truly had a diagnosis, how serious it was, and what, if anything, they should do next. Emotional responses ranged from passive trust to frustration and anxiety: “*I kind of feel angry… I don’t know really what else I can say about it; it’s just the whole information - no support*,* no nothing*” (Patient 4).

Uncertainty and reassurance also shaped how patients engaged with services afterwards. For some, reassurance discouraged further enquiry: “*They are nae worrying about it… so I just think*,* well what’s the point in asking?*” (Patient 10). Others recognised their own hesitation: “*I haven’t really had it explained to me… but to be honest*,* that’s partly my problem*,* I haven’t really asked enough*” (Patient 19). Information was often contingent on active questioning: “*If I hadn’t asked what it [a blood test] was for*,* I wouldn’t have been told*” (Patient 15). For some, more detail encouraged self-management - “*If I get that wee bit more information*,* then I can make adjustments*” (Patient 28). Professionals linked these patterns to social context. In deprived areas, patients were perceived as less likely to attend and more likely to present late: “*These patients with poor socioeconomic status… they’ll also have all of the comorbidities… so your non-attenders will go higher*” (GP 5). Engagement was often framed as personal responsibility: “*They do get another recall… but we don’t go about texting and saying they have not come*” (GP 6). Several professionals expressed frustration at these limits: “*We can only educate and invite them in as much as we can… apart from going to their door and dragging them in*,* I’m not really too sure what else you can do*” (GP 4).

### Navigating kidney disease-related care and support

Navigating kidney-disease-related care was often contingent not only on clinical need but on the practical and social resources available to patients. Some struggled with the cost of even basic travel: “*I don’t drive or anything like that*,* so I usually just walk or I get the bus. I can’t afford to travel by bus*,* I normally just have to walk*” (Patient 9). Others relied on friends or careful budgeting: “*My friend drives me about*,* so it makes it easier*” (Patient 5); “*Appointments are made a month in advance*,* so that gives me enough time to leave money aside*” (Patient 6). Attending appointments could itself be costly and difficult: “*I dinna hae [don’t have] the money to put petrol in*.” (Patient 10). For those in work, appointments and blood tests required trade-offs: “*I may need to take time off… that means I either lose pay or annual leave*” (Patient 4), though some had more support: *“I’m very grateful… they are aware that I’m likely to be absent from work*” (Patient 1). Patients also described variation in practical and emotional support around appointments. Some prepared in advance and brought a relative: “*I normally write doon whit [down what] I want to ask… I like [my daughter] to be there*” (Patient 6). Others went alone: “*Usually on my own… I don’t think I ever have [had anyone with me]*” (Patient 4).

Financial strain compounded the difficulties of navigating care. “*The benefits I get, it’s never enough to get me by a whole month… I have to budget a lot*” (Patient 31). For some, deprivation was severe: “*I’m only living off ten pound a month… I have to go down to the foodbanks. They give me salty foods, so I have no option but to eat them*” (Patient 9). Applications for welfare benefits were described as emotionally draining and often unsuccessful, with patients doubting whether kidney disease even qualified as a disability: “*Kidney disease doesn’t fall into that [disability] category*” (Patient 9). Professionals observed how poverty directly undermined medical advice: “*He eats what is given [by the foodbank]… tinned food that’s sugar-laden, salt-laden*” (GP 11). Healthcare professionals also reflected that people living in deprived circumstances often faced complex social and health problems that outweighed kidney care priorities: “*So if I was to ask them what was important to them, managing their chronic kidney disease is probably not even on the radar*” (Pharmacist 1).

Confidence in navigating health systems reflected how clearly information was provided and understood. Some experienced referrals as abrupt or poorly explained: “*I think I was just referred… it just happened*” (Patient 31). Patients under hospital follow-up reported clearer pathways: “*I call 111… If they advise me go to the hospital… I do that*” (Patient 18). Those managed mainly in primary care were more hesitant, contacting the GP only if symptoms were present or burdensome: “*I wouldn’t be phoning the doctor just to discuss the fact that I’m feeling anxious*” (Patient 13). In the absence of guidance, some turned to the internet and felt overwhelmed: “*You start the information gathering process and quickly learn… you don’t know what is actually right or wrong*” (Patient 22). Professionals recognised these barriers, noting inconsistent information provision. Leaflets remained the default, particularly for people who did not go online: “*A lot of our patients don’t go online… they prefer written things*” (GP 1). Yet CKD-specific resources were scarce: “*We have lots of leaflets on diabetes… but I don’t think we do have any [for kidneys]*” (Nurse 3). Long, generic leaflets were considered unhelpful (GP 5). NHS websites were sometimes signposted, but professionals doubted their reach: “*There might be stuff online… but I don’t know… there’s certainly a massive lack of awareness*” (GP 5). In multilingual contexts, reliance on interpreters was routine: “*Most of the patients that come in will come in with some sort of interpreter… or we just have one in-house on the phones*,* so we have adapted*” (GP 5).

Across accounts, the availability of structured psychosocial and wider support - such as link workers or social prescribing - was uneven. Rural general practices sometimes reported more consistent access than urban ones, where waiting times were longer and eligibility was narrower. Some patients were unsure what help existed: “*Just help with taxi fares or a transport pick-up… I’m not sure if it happens here*” (Patient 6). Others drew on charities or peer groups for advice and connection: “*I was made aware of a couple of charities… that were quite good for insights and support groups*” (Patient 18); “*There’s a community there… people going through similar things that I can ask*” (Patient 1). Clinicians recognised the contribution of third-sector organisations but found them difficult to keep track of: “*There are wonderful third-sector organisations… but it’s very hard to keep your finger on the pulse… funding changes*,* times change*” (GP 13). For underserved groups, the challenge was not only whether services existed but whether they were reachable in practice: “*How do they get that education… or even be able to access it?*” (Nurse 2).

### Permeability of primary care healthcare services

Because early CKD care is largely delivered through routine primary care rather than specialist renal services, the permeability of general primary care systems strongly shaped how participants accessed and experienced kidney-related care. For many patients, getting an appointment felt difficult unless initiated by the practice: “*To get an appointment on your own is very difficult… but if they want you down for something*,* they’ll fit you in*” (Patient 15). Rigid scheduling often clashed with everyday life. One patient working in childcare explained: “*I work in a nursery and we are asked… that we try and pick times that are outwith school hours*,* which is not always easy*” (Patient 13). By contrast, blood test appointments with nurses were described as easier: “*It is relatively easy to book an appointment… to see the nurse*” (Patient 22). Professionals confirmed that service design and timing shaped who could attend. Annual health checks were hard to fit around work: “*They find it hard in practice because of the inflexibility around opening times*” (Pharmacist 2). Others expected same-day access and were frustrated by the limited number of urgent slots: “*If you’re not well*,* you expect an appointment… there is often a mismatch between patient expectation and what is available*” (GP 11).

The medical reception interface was a consistent point of tension. Patients disliked sharing sensitive details with non-clinical staff: “*Fit [what] I find quite disillusionin’ [disillusioning] is when you phone up and the receptionist wants to ken yer life [know everything about your problem]… I’ll tell the doctor what’s wrong with me… but I’m nae tellin [I’m not telling you] you because you’re the receptionist*” (Patient 2). Professionals also described reception staff as gatekeepers, with practices experimenting with re-labelling the role as “*care co-ordinator*” (Nurse 5). Access was further shaped by housing and digital precarity. Frequent house moves or neighbourhood instability meant appointment letters sometimes never arrived: “*People sometimes move house quite a lot… their mail goes missing… they might not be at that house because they’ve moved*” (GP 14). Text-based reminders helped some, but for others, access was as fragile as a phone top-up: “*They might not be able to receive the text message because they’ve ran out of credits that week… they don’t have any money on their phone*” (GP 14).

Geography and infrastructure also influenced permeability. Accounts from both patients and professionals illustrated how access was uneven and continually negotiated. Some living in small villages reported easy access: “*My doctor’s surgery… answer very*,* very quickly. I think it’s because it’s a very small village - we are really lucky*” (Patient 15). Others in rural areas described physical and communication barriers: “*We get flooded roads*,* and sometimes we just don’t get our letters… it would be a nuisance if I received a letter late and had missed it through no fault of my own*” (Patient 18). Clinicians echoed this, noting patient resistance when asked to travel to off-site hubs: “*You’re essentially telling them… ‘Up you go to xxx or down you go into the centre of town’… they’re like*,* ‘Well*,* I’m at a GP because I’m in a zone. Why are you moving me out of zone?*’” (GP 7). In these cases, what appeared to be small system efficiencies often created new barriers for those already managing complex lives. When patients did not attend, clinicians described a spectrum of follow-up work, from multiple calls and texts to, in high-risk cases, police welfare checks: “*If it’s a high-risk patient*,* then we’ll often go an extra mile to track them down… might even get the police to go and see them*” (GP 1). Small rural surgeries were sometimes able to respond more flexibly, while inner-city practices struggled under stretched appointment systems.

### Adjudications of candidacy by health professionals

What mattered most to patients in professionals’ adjudications was being listened to, taken seriously, and given clear explanations - qualities most often experienced in hospital-based nephrology clinics. These settings allowed longer more conversational appointments where professionals validated concerns and offered reassurance: “*The doctor asks me open-ended questions and gives me the chance to explain how I’m feeling*” (Patient 1). Early reassurance and proactive action strengthened confidence: “*He automatically went to speak to a professional in the kidney field*” (Patient 13). Clear explanations and reliable follow-up further reinforced trust: “*My consultant will explain… then I’ll get a letter and a full explanation in the post*” (Patient 1). Even small acts of explanation mattered: “*Rather than just saying your kidney function is X… reassure that there are support tools out there*” (Patient 1). By contrast, these qualities were often missing in primary care, where short appointments limited discussion and left patients uncertain: *“I just really wished the GP… if they’d maybe explained what it was that was raised and how it can affect my bodily function”* (Patient 15).

Others described encounters that felt cursory or alienating, particularly for those without the perceived literacy, confidence or support to question judgements. Some experienced care as “*a box-ticking exercise… I couldn’t ask any questions*” (Patient 1). For patients with multimorbidity or mobility challenges, time-limited appointments in general practices amplified barriers: “*By the time I get to the room… it’s time for me to leave*” (Patient 21). Blunt communication, even when accurate, could feel distancing: “*The doctor said to me that*,* yeah*,* he would expect the kidney function that he’d seen in my test results*,* would expect a seventy-year-old man to have those results. I felt like that was quite blunt*” (Patient 1). Others reported little or no explanation: “*I just need somebody to explain what’s wrong with me… plus advice on how to delay this kidney infection or whatever it is*” (Patient 4). Silence was often interpreted as inaction: “*If there was something that could be done about it*,* they would have said something*” (Patient 14), implying that recognition frequently occurred only once the disease had already progressed. For some, silence itself became a judgement, interpreted as a quiet signal that nothing more could be offered: “*If there was something that could be done about it*,* they would have said something*” (Patient 14).

While for patients, adjudication was about tone, explanation and respect shaping their sense of being taken seriously, professionals described it as more about judging what patients could realistically manage in the context of deprivation, multimorbidity and difficult life circumstances. In these settings, clinicians often took on more of the follow-up work themselves, anticipating that patients might struggle to do so. As one GP explained: “*You won’t leave it with the patient to follow something up… a lot of the time you’ll anticipate that and be a bit proactive at our end… you try to put more nets in place for the patient*” (GP 1). Instead of expecting patients to self-manage, practices phoned repeatedly, wrote extra instructions on prescriptions, or involved carers and pharmacists. “*They can’t differentiate… they wouldn’t be able to tell you the difference between a lot of their medications*” (GP 1). “*It always takes a bit of detective work*,” same GP shared, describing a patient whose care was coordinated closely with his wife; when no carer was present, things were “*much more chaotic*” (GP 1). In some accounts, better-resourced teams were described as having scope for proactive recall and tailored plans, whereas in thinner systems, more responsibility appeared to rest with patients.

These adjudications also extended to lifestyle change. Professionals recognised the impact of deprivation and sought to provide personalised care that prioritised patient needs. They also acknowledged that some patients might achieve little change due to complex social and health circumstances. Clinicians contrasted the generic, instruction-based approach often used in routine reviews with more responsive, motivational conversations: *“You don’t want to be doing everything at once… but maybe have a conversation about*,* ‘If there’s one thing you could change… what would it be?’*” (GP 7). Even small gains were treated as meaningful: “*Just getting the weight down by a couple of kilos… that’s a success*” (Nurse 3). Yet CKD was seen as harder to adjudicate than diabetes or hypertension because there was little visible improvement to offer. As one GP put it, “*You have to make these changes not for positive improvement*,* but just to make things less bad*” (GP 4). Another described the difficulty of patient expectations: “*What can I do about that*,* doctor?*” to which the honest but unsatisfying reply was, “*Keep taking those pills… and hope for the best*” (GP 5). Where continuity allowed, clinicians described more scope to revisit, calibrate, and encourage.

### Offers and resistance to kidney care

In primary care, patient participants reported experiencing routine kidney monitoring as a formality rather than meaningful care. Annual reviews were described as administrative obligations, detached from everyday life with a long-term condition. “*Every time you go… you have to bring a urine sample… I don’t think I’ve received any [kidney] care for anything like that*” (Patient 12). Others wanted more dialogue: “*I just think it needs to be a little bit more personalised… there’s no real chat with a doctor unless you are ill-ill*” (Patient 13). Professionals confirmed that offers of care were not fixed but shaped locally. Some practices held back appointment slots for working patients or those who struggled to get through on the day: “*Patients can pre-book appointments*,* but we try and reserve those for people who are working or who have got a genuine reason why they couldn’t phone on the day*” (GP 13). For patients with complex needs, double appointments were offered informally: “*Anyone that is vulnerable or has a lot going on*,* we give them double appointments*” (GP 13). Such offers depended on local knowledge and staff discretion rather than formal policy, and patients noticed the difference between places where such flexibility existed and places where it did not.

Communication of results revealed further contrasts. Patients in renal clinics reported clear explanation and choice: “*They’ve always explained it to me… they ask you if you’d like to know*” (Patient 18). In primary care, results were described as opaque or delegated: “*They just said that this level is slightly off… there’s been no conversation about it*” (Patient 15). Being asked to call reception was a source of frustration: “*Why on earth am I phoning you for results? Surely somebody medically qualified should be answering the questions*” (Patient 11). Professionals acknowledged these problems and described informal workarounds. One nurse annotated blood results with ranges and CKD stages to support understanding: “*I write next to it what the normals are… and the stages of it*” (Nurse 5). She sometimes offered her email to patients who found it hard to get through by phone. “*Trying to get through to us can be quite difficult… so they can always e-mail in*” (Nurse 5). These adjustments made care more tangible, but they were piecemeal and dependent on individual initiative; in teams with fewer staff or higher turnover, such extras were harder to sustain.

Self-management support was another point where offers and resistance emerged in primary care. Patients often felt guidance was lacking: *“They’ve never told me anything like that. ‘You shouldn’t drink that or you shouldn’t eat that’… they’ve never discussed anything like that with me”* (Patient 10). Others valued written resources as something to return to after consultations: *“You leave*,* and that’s when you’ve got all the questions… having something you can read at your leisure is a definite plus”* (Patient 13). Yet for those with limited literacy, written materials were of little use: “*It’s more difficult for me to read by myself and not fully understand… there’s no one to ask what it’s talking about”* (Patient 31). Professionals described repeatedly revisiting lifestyle advice in the hope of catching patients at the right moment: “*We re-broach everything… just trying to engage people”* (Nurse 3). Disclosure also varied with context - *“They’ve been at the GP*,* not necessarily told the truth*,* and then they come to the nurse and it all comes out”* (Nurse 3). Where continuity and nurse-led reviews were stable, patients described more usable offers; where staffing and access were less stable, offers were thinner and resistance seemingly greater.

Continuity was a persistent concern for patients who described seeing different GPs at each visit: “*Every time you go it’s some different doctor… you’ve got to go through the same story every time*” (Patient 7). Fragmentation disrupted trust and sometimes created risk: “*The antibiotics that I was given aren’t safe for kidneys… they actually cause more damage*” (Patient 9). Missed follow-up compounded this: “*’You’ve got high levels of protein’… and to be honest*,* I’ve not heard anything since*” (Patient 8). Some responded with withdrawal: “*I’ve got so little faith in my GP… to go back just feels slightly pointless*” (Patient 23). Professionals acknowledged the unevenness of recall and management of missingness: “*Every practice does it slightly differently… sometimes nothing happens*,* they just get invited again the following year*” (GP 9). Pharmacists sometimes picked up gaps in reviews or used prescriptions to encourage attendance by providing a limited supply of medication: “*I’ll reauthorise it so they can get six months’ worth… and then after that I really do need them to attend for bloods*” (Pharmacist 1). In some health boards, these systems were reportedly more consistent; elsewhere, they relied on individual clinicians and workarounds.

Despite frustration and withdrawal, many patients continued to engage with their care through routine monitoring, medication and lifestyle adjustments. “*I have to get a check… to continue giving me my prescription*” (Patient 15). “*I look for a really healthy diet now… that’s all I’m really doing*” (Patient 4). “*My morning always starts off with tablets… they’re trying to control my blood pressure to control the kidney function*” (Patient 1). Yet professionals also noted that some patients resisted offers, not from disinterest but from concern about legitimacy: “*People don’t like to bother doctors or their practice unless they feel really unwell… I hear that all the time: ‘I didn’t want to bother you because I know how busy you are. It didn’t seem important*,* I feel okay*’” (GP 14). Professionals also highlighted that transportation difficulties and financial constraints often act as barriers to patient engagement: “*We’ve got patients in our practice who can’t afford the bus to come down… they can’t afford the taxi*” (GP 4). Accounts also hinted at variation, with some participants describing steadier staffing and clearer recall as enabling engagement, while in stretched urban practices, others spoke of holding back, judging their needs did not warrant taking up scarce slots.

### Operating conditions shaping candidacy in kidney care

For patients living with CKD in areas of deprivation, access to care was shaped not only by their own efforts but by the conditions under which services operated - the timing of appointments, the availability of staff, and the responsiveness of follow-up. Routine monitoring in primary care was often experienced as procedural, with responsibility for contact left unclear: “*I wasn’t given any kind of information on who I can go to*” (Patient 9). Appointments were often too short to allow meaningful dialogue, with participants expressing a preference for longer consultations: “*A twenty-minute appointment… so that you’ve got time to ask questions*” (Patient 4). Patients also described struggling to secure face-to-face consultations: “*They won’t actually physically see you*” (Patient 4). Patients often acknowledged system pressures - “*They are probably requiring more staff*,* extra resources*” (Patient 16) - but recognition of strain did not reduce the frustration of inaccessibility. Still, not all encounters were negative. Pharmacists were seen as approachable and responsive: “*The pharmacist is really good… she will come and speak to you*” (Patient 4). National policy also mattered. The removal of prescription charges was described not just as convenience but as an enabler of adherence: “*Since the government made prescriptions free… that doesn’t affect me getting my medication at all*” (Patient 7). For others, digital tools were helpful: *“We’re very lucky… they do respond within a day to this form”* (Patient 15). Automated text reminders were welcomed by one interviewee: “*They give me a text two days beforehand*” (Patient 6). Some contrasted inner-city practices as harder to access than smaller rural surgeries, hinting at how local context shaped follow-up.

Healthcare professionals described the same conditions from the vantage point of constraint. In deprived areas, shortages of staff and services were repeatedly cited: “*On the other side of practices*,* being in special measures*,* not having enough staff. So*,* you have a whole lot of different structural things that are going on*” (GP 9). As another GP put it: “*We’re all trying our best to do the best for everyone*,* but it just doesn’t feel like there’s enough resource available to get round everyone… we probably are leaving some people behind*” (GP 7). Even seemingly small service losses - such as no longer loaning blood pressure monitors or the absence of district nurses or social care workers - were seen to have a disproportionate impact on patients with multimorbidity. “*I think*,* inevitably*,* we are leaving behind some. We know we are… it’s those who are in the most deprived sections of society that are getting the least good healthcare*” (GP 4). Digital infrastructure was described as patchy. Apps were widely used for asthma and diabetes, but CKD was said to be “*very niche compared to the wider market for it*” (GP 5). Language barriers added another layer: “*It’s difficult for some people who English is not their first language to register at the practice… so they’re trying to make that a bit more accessible*” (GP 1). What for some was framed as innovation or efficiency was, for others, experienced as exclusion.

Policy shifts were also seen as influential. Several GPs pointed to the removal of CKD from the Quality and Outcomes Framework (QoF) as a turning point: “*eGFR and lab analysis came around the same point as the introduction of QoF in 2003–2004… It was incentivised and became an important target… now it’s been removed from the GP contract in Scotland*” (GP 9). Without this incentive, recall systems became harder to sustain, especially in practices already under strain. Wider support also varied by place. “*It’s better in the shire… social care will routinely be involved… but we don’t have that*,* unfortunately*,* in the city*” (GP 1). Where services did exist, they were not always straightforward to use. One team reported that they also practised as social prescribers: “*We don’t have an actual clinic. We just have a group of us… nurse*,* GP*,* pharmacist… so we made a folder on the drive with Sleepio and things like that… so there’s different headings for each topic*” (Nurse 3). Communication with renal teams was likewise uneven. Some valued comprehensive letters: “*Their letters are really comprehensive… they flag up actions for the GP at the start*,* which is really helpful*” (GP 1). Others described professional-to-professional communication systems that felt anonymous: “*We’ve got this alleged system… where you’re supposed to write an advice letter… I’ve never done that with renal. There’s this feeling of anonymisation and not knowing… you might recognise names*,* but you don’t know who people are*” (GP 4). In short, the links between primary and secondary care were fragile and variable.

Within practices, CKD work was spread across nurses, pharmacists and advanced nurse practitioners. “*Most of the meaningful interventions I’d say would probably be non-GP these days*” (GP 9). While efficient, this sometimes clashed with patient expectations: “*They felt that they were totally being fobbed off… they weren’t very satisfied sometimes when they walked in and I was there*” (Pharmacist 2). Professionals noted that CKD care often relied on informal champions: “*You really need somebody that’s going to champion it… whether that’s diabetes or CKD… then it becomes a wee bit by osmosis*,* influencing others’ practices*” (Pharmacist 1). Without such champions, CKD was at risk of being overshadowed by other conditions. Access to training was also described as limited: “*We don’t get PLT [Protected Learning Time] any more… even out-of-hours things you often can’t attend because you’re still at work*” (GP 5). Preventive care was frequently squeezed: *“I get ten minutes for each of my patients… there’s so much I would love to help them with… but I’ve got to move on”* (GP 11). Pharmacists, with more discretion, sometimes carved out longer reviews: *“Maybe I’m a wee bit better at being able to protect my time… I can create fifteen or thirty minutes to review somebody if they are really complicated”* (Pharmacist 1). Considering system and local challenges in primary care, professionals highlighted that current healthcare provision fails to meet the needs of patients with deprived circumstances.

### Precarity shaping the conditions for early kidney care

Participants’ accounts showed that socioeconomic and emotional precarity often predated awareness of kidney disease and shaped how its later identification and management were negotiated in practice. Ongoing pressures related to work, finances, housing, and mental health influenced participants’ capacity to prioritise kidney health, attend appointments, seek clarification, and remain engaged with services, forming the context in which candidacy for early kidney care developed. Physical fatigue, chronic pain, and declining function disrupted employment from Stage 4 onwards, though some Stage 3 participants also described early signs that affected their ability to work. Many reduced their hours, going from full-time to part-time: “*I used to work full-time … but again*,* that was too much*,* so I had to come down to two mornings a week*” (Patient 10). Another described the exhausting cycle of justifying kidney health needs to their employer: “*three occupational health reports… letters from my doctors… there’s only so much I can do*” (Patient 28). Concerns about future employment arose even in Stage 3: “*I had no option but to hand in my notice… and I do worry… it may also affect that next job as well*” (Patient 9). Financial strain was a recurring theme, compounded by low income and, in some cases, unstable housing. This strain became especially pronounced in Stage 4 CKD, with one participant describing a cascading collapse of support: “*All these things just happened and crashed from underneath me… I was homeless… I had no job… and myself and my partner did split up*,* so I was away from my children*” (Patient 37). Even earlier in the disease course, the cost of self-management - attending appointments, following dietary advice, maintaining routines - was financially burdensome. As one participant put it: “*Kidney disease financially is expensive… making things [food] from scratch can be more costly*” (Patient 9). Multiple health issues compounded the burden: “*trying to keep on top of*” health issues while “*always on the go*” (Patient 8).

Emotional strain was also a consistent background feature across accounts, described not as occasional distress but as persistent low mood, frustration, and mental fatigue - “*I’ve found myself to be a little bit more moody and cranky… I think it’s just mainly to do with the mental health that comes with the kidney disease*” (Patient 9). Others described a shift of baseline: “*My moods in general*,* I’m just low - constantly low mood*” (Patient 28). This was particularly common among those managing multiple conditions alongside social or financial pressures. Some withheld how they felt to avoid worrying others: “*I keep myself to myself… I don’t put my health issues onto my children*” (Patient 21). Others noticed changes in how they related to people: “*Very short-tempered. Tetchy. Feel as if I’m arguing with people quite a lot*” (Patient 28).

In people with severely decreased kidney function or established kidney failure, the emotional and physical demands of illness took a toll on intimate relationships: “*I just can’t get out of my bed… I feel sorry for my husband*” (Patient 10). At that point, some described reaching a crisis point: “*I was suicidal. I looked at throwing myself in the Aberdeen harbour… I didn’t know where to turn*” (Patient 37). That same patient participant later noted receiving support through primary care: “*I was put back on antidepressants to help my suicidal thoughts… and now I feel a lot stronger*” (Patient 37). Others sought mental health support outside the NHS: “*I do see a counsellor on a weekly basis… I talk to them about how I’m feeling mentally*” (Patient 1).

## Discussion

### Summary of findings

Consistent with prior quantitative evidence of later CKD identification in deprived areas [43], our qualitative findings show that, for working-age adults, “early” kidney care is often structurally late - typically initiated only once disease is already advanced and personal resources are depleted - thereby limiting capacity to engage in preventive action. Although incidental detection is characteristic of early CKD, it is explained briefly, if at all, leaving patients uncertain about its seriousness and what action is required. Conversations about kidney health vary in clarity and tone, shaping whether people understand themselves as needing ongoing care or interpret reassurance as closure. Many struggle to navigate complex and fragmented systems, where appointments, referrals, and follow-up depend on persistence, timing, and personal means. Professional assessments are similarly constrained by uncertainty, limited support, and the constant need to prioritise amid multimorbidity and system strain. Offers of care are uneven, sometimes softened or deferred, and their usefulness depends on continuity and relationships rather than standardised processes. Much care relies on discretionary effort - extra calls, annotated results, and active test-chasing - while community resources such as link workers and voluntary organisations bridge the gap between clinical advice and daily life. While these efforts often sustain care under pressure, they also render access uneven, as follow-up becomes contingent on individual initiative and local capacity rather than embedded systems.

### Interpretation: candidacy under constraint

Viewed through the Candidacy Framework [16], whether and how people enter early kidney care depends on continual negotiation with services, shaped by time, trust, and capacity. Eligibility and engagement depend on how people’s daily lives align - or fail to align - with the organisation of primary care [13, 48]. In this context, late detection and inconsistent follow-up reflect not a lack of motivation but the structural conditions that constrain recognition, help-seeking, and sustained engagement. As one participant reflected, “*They are nae worrying about it… so …well what’s the point in asking?*” (Patient 10). This illustrates what we term candidacy under constraint - where patients internalise limited professional concern as a cue that their condition is not a priority, disengaging not from apathy but from an awareness of system strain. Both patients and professionals work to sustain care within these constraints, often relying on discretionary effort and local relationships to bridge systemic gaps. Together, these local negotiations reveal the broader logic of the Inverse Care Law [11], where those with the greatest need face the most limited capacity for timely care.

Seen through the lens of candidacy as socially structured access, relational mechanisms - trust, continuity and communication - are not optional interpersonal features but core conditions shaping service permeability [31, 46]. These mechanisms operate within wider structural constraints, where workforce capacity, organisational priorities and socioeconomic conditions shape both daily life and service delivery. Recent Scottish evidence indicates that such pressures persist despite policy commitments to equity [5]. In practice, primary care capacity remains a key constraint, with staffing pressures and turnover narrowing opportunities for testing, follow-up and timely diagnosis [2]. National initiatives aimed at addressing social and health inequalities will depend on sustained workforce time and community capacity to translate policy into practice.

Our study focused on experiences of living in areas of multiple deprivation as a key structural determinant shaping access to care. While demographic information, including gender, was collected, individual characteristics were not explored as primary analytic categories, and no consistent gender-specific patterns were identified across themes. However, gender has been identified as an important dimension shaping access to healthcare in type 2 diabetes - a condition associated with increased risk of CKD [10]. Although this evidence is drawn from a different clinical context and healthcare setting, it highlights how gender may interact with socioeconomic and structural conditions to influence engagement with care. In our study, participants described substantial psychosocial demands, including emotional strain and competing life pressures, which may also be shaped by gender, even where not explicitly articulated. Previous qualitative studies applying a candidacy lens have also demonstrated how gender intersects with social context to shape perceptions of risk and engagement with care [3]. Future research could apply an explicitly intersectional lens to examine how gender interacts with structural conditions, such as socioeconomic deprivation, age, and access to services, in shaping candidacy for early kidney care, and may help inform more tailored and person-centred approaches to service design.

### Implications: equitable early kidney care

These findings help explain why kidney health remains under-prioritised within non-communicable disease strategies. At a population level, early stages of kidney disease have limited public health visibility, reflected in routine data sources and their relative absence from national dashboards. At a system level, kidney disease lacks the enhanced services and incentives afforded to conditions such as diabetes and cardiovascular disease in primary care. At an individual level, early CKD is largely asymptomatic or characterised by non-specific symptoms, further limiting recognition and salience. Together, this “triple invisibility”, at population, system, and symptom levels, weakens candidacy for early kidney care and constrains preventive action.

Viewing CKD as a biomedical-psychosocial juncture reframes early kidney care as both detection and action. The question is not only how to detect disease earlier, but how to create the conditions that make early action possible. Ensuring capacity for early kidney care within an overstretched health system is essential to prevent inequities from widening. This requires protected opportunities for early detection, timely communication, and structured follow-up before disease progression limits prevention. It also requires institutional recognition that time, continuity, and trust are core conditions for equitable care rather than optional extras. Action across primary and community settings should safeguard consultation time, ensure that results are communicated clearly with agreed next steps, and embed navigation for social and practical needs such as transport, employment, housing, digital access, and psychosocial support. Although kidney function tests are among the most frequently performed investigations in UK primary care [25], our findings highlight ongoing challenges in how patients and professionals interpret and act on results. Clear framing of blood and urine ing within annual reviews - emphasising kidney health and shared next steps - may reduce softened or protective communication practices that inadvertently contribute to partial or non-disclosure of CKD [14, 15]. Strengthening these temporal and relational aspects of care may help early detection translate into sustained engagement and reinforce prevention as a shared, enduring capacity [31]. Future research should build on this work through co-design with people affected by CKD, primary care teams, and community partners to develop and test integrated models of care that embed appropriately framed conversations about kidney health into routine practice, address the service constraints identified here, and enable earlier and more equitable action.

These findings also connect to the emerging concept of cardiovascular–kidney–metabolic (CKM) syndrome, which recognises cardiovascular disease, chronic kidney disease, and metabolic disorders as part of a linked continuum of biological and social risk [39]. The CKM model emphasises staged risk assessment and prevention at the earliest identifiable stages of cardiometabolic risk, incorporating kidney function and albuminuria testing within integrated care pathways. Importantly, it situates social and structural determinants of health - including socioeconomic position, stress exposure, and access to care - as part of the causal pathway rather than as external context. Our findings complement this perspective by clarifying what translating integrated risk recognition into sustained preventive action would require in practice, particularly in deprived areas. Achieving the CKM ambition will depend on addressing the organisational and relational constraints identified here - limited time, fragile continuity, and uneven community capacity - through workforce models that integrate kidney health across routine cardiovascular and metabolic encounters. In doing so, prevention can move from aspiration to implementation, enabling earlier and more equitable action within real-world service conditions.

### Implications: theory

Our findings extend recent refinements of candidacy in use and scope [30, 33] by showing that, in early CKD, clinical rather than patient-led identification initiates the candidacy process, requiring an empirically adapted ordering of the early domains to reflect how participants encountered care in practice. We further show that socioeconomic precarity and organisational strain are embedded within, and operate across, candidacy domains, structuring capacity to appear in services, navigate care pathways, and engage with offers of care. These processes were bidirectional and contingent on disease severity: while precarity shaped candidacy most strongly before and at early recognition, progression of CKD often intensified existing socioeconomic and emotional strain, further constraining engagement. Multimorbidity additionally distributed attention and priority across conditions, shaping how kidney disease was interpreted and adjudicated by professionals. Taken together, these insights complement existing work by positioning candidacy more explicitly within the structural determinants shaping access to asymptomatic and clinically mediated conditions.

### Strengths and limitations

This study’s key strengths include the multi-site design across three Scottish boards and the focus on a working-age and lived experience of area deprivation (i.e. a seldom-heard population); this is complemented by accessibility-minded recruitment (easy-read materials, flexible modes), dual perspectives (patients and professionals), and theory-informed analysis underpinned by substantive patient, public and professional engagement.

The present qualitative study did not sample across the full socioeconomic gradient, which would have enabled systematic examination of how structural mechanisms operate across levels of socioeconomic advantage and disadvantage. However, previous quantitative analysis using linked population-level data has demonstrated clear socioeconomic gradients in CKD-related outcomes, with greater mortality and poorer health indicators among people experiencing higher levels of deprivation [43]. The current study, therefore, focused on an in-depth exploration of how these inequalities are experienced and negotiated in practice among those known, from linked population-level data, to face constrained access to care, reflecting a complementary qualitative emphasis on mechanism and process.

This study is situated within the context of Scotland’s NHS, which represents a methodological consideration; however, convergence with UK-wide evidence on primary care during the austerity era [17] supports the transferability of the findings. In addition, participants were sampled according to eGFR-based CKD staging rather than full KDIGO risk classification, incorporating albuminuria, which may have provided additional nuance in how CKD is considered and discussed for individuals with higher albuminuria despite mild or moderately reduced kidney function. However, as albuminuria is not consistently recorded in primary care, sampling on this basis would likely have introduced systematic exclusion and difficult-to-interpret accounts, undermining analytic coherence.

## Conclusions

Early kidney care in communities experiencing deprivation represents a structurally constrained preventive window in which biological risk intersects with lived social conditions shaping opportunities for action. In the absence of population, system and symptom visibility, prevention becomes dependent on discretionary effort and individual capacity at the point when intervention is most effective. Access and engagement therefore emerge as processes of candidacy negotiated within limited time, continuity and system capacity, revealing how organisational and social structures, rather than individual motivation, reproduce inequities even within universal healthcare. Strengthening equitable prevention requires community-linked primary care models that embed social and structural determinants within care design, consistent with integrated cardiovascular-kidney-metabolic approaches that situate prevention and inequity within the causal pathway.

## Supplementary Information

Below is the link to the electronic supplementary material.

Supplementary Material 1

Supplementary Material 2

Supplementary Material 3

Supplementary Material 4

## Data Availability

A complete qualitative coding framework with a hierarchical coding tree is available via the University of Aberdeen PURE repository.

## Declarations

### Authors’ information

MRD is an Advanced Research Fellow and qualitative health researcher specialising in health services research and equity-focused implementation science; she is also an accredited community-based counsellor. BK is a Postdoctoral Researcher with a background in health psychology and expertise in qualitative health research. SS and PC are clinical academic nephrologists with a focus on population kidney health and deprivation-related inequalities. TB and BJ are academic general practitioners with expertise in multimorbidity and primary care service organisation. SB is a clinical epidemiologist specialising in chronic disease outcomes. MMa is a Professor of Medical Sociology with expertise in structural determinants of health and health inequalities. NSR and EC are academic researchers contributing to chronic disease and health services research. AH, MG, MMe and FN are Patient and Public Involvement contributors.

## Abbreviations

CKD: Chronic kidney disease
AKI: Acute kidney injury
SIMD: Scottish Index of Multiple Deprivation
eGFR: estimated glomerular filtration rate
ACR: Albumin-to-creatinine ratio
NHS: National Health Service
NRS: NHS Research Scotland
RTA: Reflexive thematic analysis
GRIPP2: Guidance for Reporting Involvement of Patients and the Public
COREQ: Consolidated criteria for reporting qualitative research
KDIGO: Kidney Disease: Improving Global Outcomes
QoF: Quality and Outcomes Framework
