# Supplementary material for: “They are nae worrying about it… so …what’s the point in asking?”: A qualitative study of candidacy for early CKD care in areas of multiple deprivation
Source: BMC Nephrol. 2026 Jun 11;27:485. doi: 10.1186/s12882-026-05084-9 (PMC13488199; doi:10.1186/s12882-026-05084-9)
Supplement: Supplementary file 1 [file 12882_2026_5084_MOESM1_ESM.docx]

**Additional file 1: KDIGO chronic kidney disease (CKD) risk heat map.**

Manuscript: “They are nae worrying about it… so …what’s the point in asking?”: a qualitative study of candidacy for early CKD care in areas of multiple deprivation

|  |  |  |  | **Albuminuria* categories**  Description and range | | |
| --- | --- | --- | --- | --- | --- | --- |
|  |  |  |  | **A1** | **A2** | **A3** |
|  |  |  |  | Normal to mildly increased | Moderately increased | Severely increased |
|  |  |  |  | <30 mg/g | 30-299 mg/g | ≥300 mg/g |
| **GFR^†^ categories (ml/min/1.73 m²)**  Description and range | **G1** | Normal to high | ≥ 90 | Screen (1) | Treat (1) | Treat & refer (3) |
|  | **G2** | Mildly decreased | 60-89 | Screen (1) | Treat (1) | Treat & refer (3) |
|  | **G3a** | Mildly to moderately decreased | 45-59 | Treat (1) | Treat (2) | Treat & refer (3) |
|  | **G3b** | Moderately to severely decreased | 30-44 | Treat (2) | Treat & refer (3) | Treat & refer (3) |
|  | **G4** | Severely decreased | 15-29 | Treat & refer (3) | Treat & refer (4+) | Treat & refer (4+) |
|  | **G5** | Kidney failure | <15 | Treat & refer (4+) | Treat & refer (4+) | Treat & refer (4+) |

|  | Low CKD risk |  | | High risk |  |
| --- | --- | --- | --- | --- | --- |
|  | Moderately increased risk |  |  | Very high risk | |

**Figure S1.1.** KDIGO^⸹^ heat map for CKD classification.

KDIGO action categories indicating recommended intensity of monitoring, treatment, and referral according to CKD risk:

Screen (1): Routine monitoring and risk assessment in primary care.

Treat (1): Optimise standard risk-factor management in primary care.

Treat (2): Initiate or intensify kidney- and cardiovascular-protective treatment with closer monitoring.

Treat and refer (3): Active treatment plus referral to nephrology, according to local pathways.

Treat and urgently refer (4+): Intensive treatment with prompt specialist nephrology care.

*Late presentation*: CKD first identified at a stage requiring treat and refer (3) or treat and urgently refer (4+).

CKD; chronic kidney disease; ^†^GFR, glomerular filtration rate (how well the kidneys are filtering waste from the blood); *Albuminuria, increased albumin (protein) in the urine (a sign that the kidneys are damaged or under strain)

⸹Adapted from Kidney Disease: Improving Global Outcomes (KDIGO) CKD Work Group. KDIGO 2024 Clinical Practice Guideline for the Evaluation and Management of Chronic Kidney Disease. Kidney Int. 2024;105(4S):S117-S314.
