# Supplementary material for: “They are nae worrying about it… so …what’s the point in asking?”: A qualitative study of candidacy for early CKD care in areas of multiple deprivation
Source: BMC Nephrol. 2026 Jun 11;27:485. doi: 10.1186/s12882-026-05084-9 (PMC13488199; doi:10.1186/s12882-026-05084-9)
Supplement: Supplementary file 2 [file 12882_2026_5084_MOESM2_ESM.docx]

**Additional file 2:** **Patient, Public and Professional Involvement (PPPI) statement.**

Manuscript: “They are nae worrying about it… so …what’s the point in asking?”: a qualitative study of candidacy for early CKD care in areas of multiple deprivation

Here, we describe these activities using the GRIPP2 Short Form framework (Staniszewska et al., 2017), which organises reporting across five key aspects: the aim of involvement, the methods through which it was enacted, the results and outcomes it generated, critical reflections on its challenges, and the impact it produced within the study.

**Aim of involvement:**
Stakeholder engagement - including patients, public partners, and professionals - was embedded throughout the project to ensure that the design, implementation, and interpretation of findings were shaped by diverse expertise and lived experience. This approach aimed to enhance the relevance, inclusivity, and equity of the study in understanding early kidney care.

**Methods used for involvement:**
The project was supported by an Advisory Group of 19 members, including nephrology consultants, general practitioners, methodology experts, public-health specialists, and a health-equity programme manager. The Implementation-STakeholder Engagement Model (I-STEM; Potthoff et al., 2023) guided the roles of professional and public stakeholders across all project phases. Structured meetings and asynchronous consultations were used to gather feedback on study design, data collection, and emerging analysis. Preliminary patient findings were presented to professional stakeholders at renal clinics and general practices, and their feedback helped refine subsequent interview topics and contextualise interpretation.

Patient and public partners worked alongside the research team throughout, following a co-productive ethos informed by Hickey et al.’s (2018) partnership model and Greenhalgh’s (2016) view of knowledge as relational and co-constructed. The PPI team comprised one co-applicant living with kidney disease and two public partners active in community organisations representing underserved and ethnically minoritised populations. They contributed to co-designing recruitment materials, consent forms, and information sheets (including video-recorded versions for accessibility) and helped pilot and refine the interview topic guide. The patient co-investigator also supported community outreach through the Grampian Kidney Patient Association and Scottish Kidney Federation.

**Outcomes of involvement:**
Patients, public partners, and professionals each influenced the study in distinct yet complementary ways. The involvement of patients and public partners improved the accessibility and cultural appropriateness of participant-facing materials, resulting in redesigned easy-read information sheets and visual consent tools. Their feedback led to more flexible recruitment processes and the inclusion of remote interview options to accommodate those managing multiple demands. Professional stakeholders helped ensure representativeness across practice settings, CKD stages, and patient demographics. During data collection, PPPI members highlighted the need to explore psychosocial strain and mental health in greater depth, prompting refinement of the interview guide. During analysis, all stakeholder groups participated in iterative interpretive discussions. Lived-experience partners validated emerging themes and encouraged interpretation through an equity lens, while professionals contributed to framing the organisational and policy contexts shaping everyday kidney care. Collectively, their input informed the conceptualisation of the “thinly supported frontier” to describe the fragility of early CKD care, ensuring that findings captured both lived and professional realities.

**Reflections:**
Engaging patients, public partners, and professionals together required sustained relational work and iterative communication. Managing differences in time availability, familiarity with research processes, and professional responsibilities demanded flexibility and sensitivity. These challenges reflected the broader PPI literature emphasising trust, reciprocity, and time investment as key conditions for meaningful involvement (Brett et al., 2014). Ensuring equitable participation also meant accommodating diverse needs, supporting contributors to navigate emotional topics, and recognising their contributions fairly. Professionals’ competing demands within overstretched systems limited their availability at times, mitigated through asynchronous engagement and concise feedback processes.

**Impact of involvement:**
Engaging multiple stakeholder groups shaped both the process and the substance of this research. Their combined insights strengthened ethical integrity, deepened understanding of lived precarity, and enhanced analytic richness. Professional stakeholders contextualised patient and public experiences within organisational and policy constraints, while lived-experience contributors grounded the analysis in the realities of deprivation, trust, and access. The resulting synthesis allowed the study to reframe early kidney care as a socially and temporally produced practice rather than a purely biomedical stage. These outcomes are consistent with evidence that meaningful and inclusive engagement improves the quality, relevance, and legitimacy of health research (Staniszewska et al., 2017; Brett et al., 2014).

**References**

Brett J, Staniszewska S, Mockford C, Herron-Marx S, Hughes J, Tysall C, Suleman R. A systematic review of the impact of patient and public involvement on health and social care research. *Health Expect*. 2014;17(5):637-650.

Greenhalgh T. Achieving research impact through co-creation in community-based health services: literature review and case study. *Milbank Q*. 2016;94(2):392-429.

Hickey G, Brearley S, Coldham T, Denegri S, Green G, Staniszewska S, Tembo D, Torok K, Turner K. Moving from tokenism to meaningful engagement: best practices in patient involvement in research. *Res Involv Engagem*. 2018;4:1-10.

Potthoff S, Presseau J, Johnston M, et al. Implementation–Stakeholder Engagement Model (I-STEM): a practical framework for involving stakeholders in implementation research. *Implement Sci*. 2023;18:23.

Staniszewska S, Brett J, Simera I, Seers K, Mockford C, Goodlad S, Altman DG, Moher D, Bell P, M’Cannon CJ, Vale C, Stewart D, Ciccone A, Denegri S, Entwistle A, Littlejohns P, Morris C, Suleman R, Thomas V, Tysall C. GRIPP2 reporting checklists: tools to improve reporting of patient and public involvement in research. *BMJ*. 2017;358:j3453.
