# Supplementary material for: “They are nae worrying about it… so …what’s the point in asking?”: A qualitative study of candidacy for early CKD care in areas of multiple deprivation
Source: BMC Nephrol. 2026 Jun 11;27:485. doi: 10.1186/s12882-026-05084-9 (PMC13488199; doi:10.1186/s12882-026-05084-9)
Supplement: Supplementary file 3 [file 12882_2026_5084_MOESM3_ESM.docx]

**Additional file 3: Participant characteristics.**

Manuscript: “They are nae worrying about it… so …what’s the point in asking?”: a qualitative study of candidacy for early CKD care in areas of multiple deprivation

Table S3.1 lists all 38 patient participants individually, with age, employment, ethnicity, and comorbidities grouped into broader categories to reduce the risk of de-anonymisation. A health board and recruitment route are retained to show the sampling context. SIMD quintile and rural-urban classification are included to capture the socioeconomic and geographical contexts of participants’ lives, which are central to understanding inequalities in kidney care.

**Table S3.1.** Participant characteristics (patients).

| **ID** | **Sex** | **Age group** | **Self-reported ethnicity** | **Employment status** | **SIMD quintile^¥^** | **Rural-urban classification^§^** | **Health board** | **Recruitment route** | **CKD stage based on (based on most recent eGFR if available)** | **Comorbidities (spontaneously self-reported during the interview)** |
| --- | --- | --- | --- | --- | --- | --- | --- | --- | --- | --- |
| Patient 1 | M | 30-39 | White British | Employed | 2 | Large Urban Areas | Lothian | Community outreach | Stage 4 (self-reported) | None reported |
| Patient 2 | M | 60-65 | White British | Long-term sick/disabled | Not available | Not available | Grampian | Primary care | Stage 3a (eGFR 56) | Cancer |
| Patient 3 | M | 60-65 | White British | Long-term sick/disabled | 2 | Large Urban Areas | Grampian | Secondary care | Stage 5 (eGFR 13) | Hypertension |
| Patient 4 | F | 60-65 | White British | Employed | 1 | Large Urban Areas | Grampian | Primary care | Stage 3a (eGFR 57) | Respiratory, Musculoskeletal |
| Patient 5 | F | 60-65 | White British | Employed (part-time) | 2 | Large Urban Areas | Grampian | Primary care | Stage 2 (eGFR >60) | Cardiovascular, Musculoskeletal |
| Patient 6 | F | 50-59 | White British | Long-term sick/disabled | 2 | Large Urban Areas | Grampian | Primary care | Stage 3a (eGFR 53) | Diabetes |
| Patient 7 | F | 40-49 | White British | Employed | 2 | Large Urban Areas | Grampian | Primary care | Stage 3b (eGFR 42) | None reported |
| Patient 8 | F | 60-65 | White British | Employed | 2 | Large Urban Areas | Grampian | Primary care | Stage 2 (eGFR >60) | Urological |
| Patient 9 | M | 30-39 | White British | Unemployed | 2 | Large Urban Areas | Grampian | Primary care | Stage 3b (eGFR 36) | Learning difficulties |
| Patient 10 | F | 60–65 | White British | Employed (part-time) | 2 | Large Urban Areas | Grampian | Primary care | Stage 4 (self-reported) | Musculoskeletal, Diabetes |
| Patient 11 | F | 60-65 | White British | Employed | 1 | Large Urban Areas | Grampian | Primary care | Stage 3a (eGFR 59) | None reported |
| Patient 12 | F | 50-59 | Black British | Employed | 2 | Large Urban Areas | Grampian | Primary care | Stage 2 (eGFR >60) | Gastrointestinal |
| Patient 13 | F | 50-59 | White British | Employed | 1 | Large Urban Areas | Grampian | Primary care | Stage 2 (eGFR 60) | None reported |
| Patient 14 | M | 60-65 | White British | Retired | 2 | Accessible Rural | Grampian | Primary care | Stage 3a (eGFR 48) | Cardiovascular |
| Patient 15 | F | 60-65 | White British | Employed (part-time) | 2 | Accessible Rural | Grampian | Primary care | Stage 3a (eGFR 56) | Musculoskeletal |
| Patient 16 | M | 50-59 | White British | Employed | 1 | Accessible Small Towns | Fife | Primary care | Stage 3a (eGFR 54) | None reported |
| Patient 17 | M | 60-65 | White British | Employed | Not available | Not available | Tayside | Secondary care | Stage 3a (eGFR 48) | Diabetes |
| Patient 18 | M | 18-29 | White other | Employed | 2 | Accessible Rural | Grampian | Secondary care | Stage 1 (eGFR>60) | None reported |
| Patient 19 | M | 60-65 | White British | Employed | 1 | Accessible Small Towns | Fife | Primary care | Stage 3a (eGFR 54) | Hypertension |
| Patient 20 | M | 30-39 | White British | Employed | 2 | Accessible Rural | Grampian | Primary care | Stage 5 (eGFR not available) | None reported |
| Patient 21 | F | 30-39 | White British | Long-term sick/disabled | 1 | Accessible Small Towns | Fife | Primary care | Stage 3a (eGFR 58) | Musculoskeletal, Haematological |
| Patient 22 | M | 50-59 | White British | Employed | 2 | Accessible Rural | Grampian | Primary care | Stage 3a (eGFR 54) | None reported |
| Patient 23 | F | 50-59 | White British | Employed | 2 | Accessible Small Towns | Greater Glasgow & Clyde | Primary care | Stage 3a (self-reported) | Hypertension |
| Patient 24 | F | 50-59 | White British | Retired | Not available | Not available | Grampian | Primary care | Stage 4 (eGFR 19) | Urological |
| Patient 25 | F | 60-65 | White Irish | Employed (part-time) | 2 | Accessible Rural | Grampian | Primary care | Stage 3b (eGFR 31) | Diabetes |
| Patient 26 | M | 50-59 | White British | Employed | 2 | Large Urban Areas | Greater Glasgow & Clyde | Primary care | Acute kidney injury | Diabetes |
| Patient 27 | F | 30-39 | White British | Employed (part-time) | 1 | Large Urban Areas | Tayside | Secondary care | Stage 3b (eGFR 32) | Hypertension, Diabetes |
| Patient 28 | M | 50-59 | White British | Employed | 1 | Large Urban Areas | Greater Glasgow & Clyde | Primary care | Stage 2 (eGFR >60) | Hypertension, Musculoskeletal |
| Patient 29 | M | 40-49 | Asian  British | Employed | 2 | Large Urban Areas | Grampian | Secondary care | Stage 2 (eGFR >60) | Diabetes |
| Patient 30 | M | 30-39 | White other | Employed (part-time) | 2 | Large Urban Areas | Tayside | Secondary care | Stage 3a (eGFR 47) | Hypertension, Diabetes |
| Patient 31 | F | 18-29 | White British | Unemployed | 1 | Other Urban Areas | Grampian | Secondary care | Stage 2 (eGFR >60) | Diabetes |
| Patient 32 | M | 60-65 | White other | Long-term sick/disabled | 1 | Large Urban Areas | Greater Glasgow & Clyde | Primary care | Stage 2 (eGFR >60) | Hypertension, Diabetes |
| Patient 33 | F | 50-59 | Black British | Long-term sick/disabled | 1 | Large Urban Areas | Greater Glasgow & Clyde | Primary care | Stage 3a (eGFR 52) | Hypertension, Diabetes |
| Patient 34 | M | 50-59 | White British | Employed | Not available | Not available | Greater Glasgow & Clyde | Primary care | Stage 3b (eGFR 34) | Diabetes |
| Patient 35 | F | 50-59 | White British | Employed | Not available | Not available | Fife | Primary care | Stage 2 (eGFR 60) | Musculoskeletal |
| Patient 36 | F | 30-39 | White British | Employed | 2 | Accessible Rural | Fife | Primary care | Not available | None reported |
| Patient 37 | M | 50-59 | White British | Unemployed | 1 | Large Urban Areas | Grampian | Secondary care | Stage 4 (eGFR 19) | Diabetes, Cardiovascular |
| Patient 38 | M | 60-65 | White British | Employed | 1 | Large Urban Areas | Grampian | Secondary care | Stage 3a (eGFR 50) | Diabetes |

**^¥^** Postcodes were entered into the SIMD 2020 tool (<https://simd.scot>) to obtain deprivation deciles and **^§^** manually cross-checked against the 2020 Scottish Government Urban-Rural Classification (<https://www.opendata.nhs.scot/dataset/urban-rural-classification>) to assign UR6 categories. The absence of SIMD and rural-urban classifications for five participants reflects their decision not to disclose postcodes directly to the research team. Postcodes had already been verified via NHS Research Scotland to establish eligibility, but disclosure to researchers was optional.

*We extracted from medical records the current CKD stage recorded by clinicians, the most recent estimated glomerular filtration rate (eGFR), and any previously recorded CKD stage where available. To minimise re-identification risk and support clarity, we report here only the most recent CKD stage, derived from the most recent eGFR in line with KDIGO GFR classification (Additional file 1). Although KDIGO refers to GFR categories (not eGGFR), kidney function is reported in clinical practice as eGFR, which is therefore used here. Participants were not recruited or stratified by albuminuria stage; albuminuria data were not collected, as they are not routinely or consistently available in primary care and were not required for the qualitative aims of the study. The analysis thus reflects kidney health information typically available to GPs rather than formal risk stratification incorporating albuminuria.

**Table S3.2.** Participant characteristics (professionals).

| **ID** | **Data collection method** | **Role** | **Health board** |
| --- | --- | --- | --- |
| GP 1 | Focus group 1 | GP | Grampian |
| GP 2 | Focus group 1 | Trainee GP | Grampian |
| GP 3 | Focus group 1 | Trainee GP | Grampian |
| Nurse 1 | Focus group 1 | Practice nurse | Grampian |
| GP 4 | Focus group 2 | GP | Grampian |
| GP 5 | Focus group 2 | GP | Grampian |
| Nurse 2 | Focus group 2 | Practice nurse | Glasgow & Clyde |
| GP 6 | Focus group 3 | GP | Grampian |
| Nurse 3 | Focus group 3 | Practice nurse | Grampian |
| GP 7 | Focus group 3 | GP | Grampian |
| GP 8 | Focus group 3 | GP | Grampian |
| Pharmacist 1 | Focus group 4 | Pharmacist | Glasgow & Clyde |
| Pharmacist 2 | Focus group 4 | Pharmacist | Glasgow & Clyde |
| GP 9 | Focus group 4 | GP | Fife |
| GP 10 | Interview | GP | Fife |
| GP 11 | Interview | GP | Grampian |
| Nurse 4 | Interview | Practice nurse | Glasgow & Clyde |
| Nurse 5 | Interview | Practice nurse | Glasgow & Clyde |
| Nurse 6 | Interview | Practice nurse | Glasgow & Clyde |
| GP 12 | Interview | GP | Grampian |
| GP 13 | Interview | GP | Glasgow & Clyde |
| GP 14 | Interview | GP | Glasgow & Clyde |
| GP 15 | Interview | GP | Grampian |
