# Supplementary material for: “They are nae worrying about it… so …what’s the point in asking?”: A qualitative study of candidacy for early CKD care in areas of multiple deprivation
Source: BMC Nephrol. 2026 Jun 11;27:485. doi: 10.1186/s12882-026-05084-9 (PMC13488199; doi:10.1186/s12882-026-05084-9)
Supplement: Supplementary file 4 [file 12882_2026_5084_MOESM4_ESM.docx]

**Additional file 4:** **Topic guides for qualitative data collection.**

Manuscript: “They are nae worrying about it… so …what’s the point in asking?”: a qualitative study of candidacy for early CKD care in areas of multiple deprivation

**Table S4.1.** Topic guide for interviews with people with kidney disease

| **Areas of questioning** | **Main questions** | **Exemplary prompts** |
| --- | --- | --- |
| **Experiences with diagnosis** | Can you talk me through the time when you first found out about your kidney problems? | - Were there clear pathways for you to receive care, or have there been times when it felt difficult to find the right service? - How did you find out? |
|  | How did you find the diagnosis and its explanation? | - How was it (the diagnosis) discussed? - Can you describe your experience when receiving information about your condition? - Is there anything that has helped you make sense of the diagnosis? - What else would you have wanted to have known? - What was your experience like communicating with healthcare professionals about your diagnosis? - How did you find the medical terms or language used during your diagnosis? |
|  | Were there any (other) challenges, if any, with making sense of the diagnosis and its relevance to your health? | - Did anything or anyone help you when you were finding out about your condition? - In what ways, if any, did you feel support was lacking during the diagnostic process? - Are there any resources or tools you came across that you found useful in making sense of your condition? |
| **Adjustments** | Has finding out about the diagnosis changed anything that you do, if at all? | - Can you describe any changes in your daily life since the diagnosis? |
|  | How has your kidney problem affected life commitments such as work, money, family, and children, if at all?  **AND IN REVERSE**  How have those life commitments affected your kidney health or the care you've received, if at all? | - In what ways…. How have your [work-related/ family/etc] responsibilities affected your health/health-related decision care? |
| **Access to care** | Can you talk me through what typically happens when you have an appointment for your kidney problem at, you’re a) practice, b) hospital clinic?  [Note: explore what happens when exceptions arise] | - Can you talk me through what happens when you arrange an appointment? Is there anything that makes this easy or difficult? |
|  |  | - Could you tell me more about how you prepare your appointments, such as going through information or having conversations with family? Is there anything that makes this easy or difficult? |
|  |  | - What is discussed at appointments? Is there anything else you would like to discuss more with your GP/nurse/hospital team? |
|  |  | - What do you think about how medicines are typically explained and given to you? Is there anything that makes this easy or difficult? |
|  |  | - How do you find scheduling tests including blood and urine tests and understanding why they were done? Is there anything that makes this easy or difficult? |
|  |  | - Can you tell me what happens when something concerns you about your kidney health, for example when you feel unwell? Is there anything that makes this easy or difficult? |
|  |  | - Can you tell me more about getting additional help or support when required? Is there anything that makes this easy or difficult? |
| **Understanding intersectionality** | Everyone's life is unique, and various aspects of our lives can shape our experiences. Without sharing anything you're uncomfortable with, can you tell me about how certain parts of your identity or personal circumstances influenced your journey with a kidney problem? Some people mention things like where they live, their financial situation, their education, any disabilities, cultural background, or other personal factors. Do any of these, or perhaps other factors, affect your experience?  [Note for the interviewer: explore also how these influences might in reverse, have contributed to developing kidney disease] | - How easy or difficult has it been for you to travel to healthcare facilities? - Have you noticed any ways that your surroundings or local resources have impacted your journey with kidney health? - In what ways, if any, has your financial situation intersected with your kidney health journey? - How has your educational background influenced your experience or understanding of your kidney problem? - Can you talk me through some instances where your cultural background specifically affected your experience with kidney health? |
| **Overall views on health and kidney care** | [Note: start by summarising things you've noticed that went well and not so well. – “So, mentioned..”]  Overall, what aspects of your kidney care went particularly well? What needs to change?  Do you perhaps have any ideas or thoughts on what a better [name an issue – preferably paraphrasing it verbatim] may look like? | - Were there any tricky situations or “deal-breakers”? - Can you share about any services or support systems that stood out to you during your care journey? - What are the most important aspects of care you received for your kidney condition? |

**Table S4.2: Topic guide for focus groups with primary care professionals**

| **Areas of questioning** | **Main questions** | **Exemplary prompts** |
| --- | --- | --- |
| **Understanding of kidney disease** | How would you describe in your practice your experience in diagnosing the care of people with CKD and AKI? | - How would you communicate a diagnosis of kidney disease (CKD, or AKI) with a patient? - What factors might affect what you discuss with them? Would you revisit this discussion or only have it at first diagnosis? - What knowledge and skills do you think are necessary for the effective diagnosis of kidney disease? |
|  | What resources or tools (e.g., guidelines, support) are available to aid in the diagnosis and management of kidney disease care, and how do you use them? | - What strategies are available to your practice to help healthcare professionals? - Could you describe the types of additional information or support you offer in your practice to patients, such as written materials, visuals, websites, consultations, or other resources, if any? |
|  | Could you share the kinds of challenges you've noticed patients facing while trying to make sense of their kidney disease and its treatment? | - Are there any adjustments that the practice or the hospital have put in place or need to be put in place to address these challenges? |
| **Multi-disciplinarity** | How does your team collaborate in the care of patients with kidney disease? | - What role do GPs, nurses, dietitians, pharmacists, admin, social prescribing and other healthcare professionals play in the care of patients with kidney disease? - How would you approach a situation where there's a concern about a patient's high risk of harm? - How do you keep track of patients in your practice to ensure you know if they are stable or deteriorating? - What would happen if a patient did not show up for an appointment or test with one of your team as part of their usual monitoring? |
|  | Could you share your experiences regarding your collaboration with renal colleagues in secondary care? | - In your perspective, what aspects of this working relationship have a positive impact on patient outcomes, and what areas do you think present opportunities for improvement? - How do you feel the type of care being provided influences the allocation of responsibility? For instance, does the responsibility shift between monitoring tests, appointments, patient-initiated consultations, or secondary care appointments? Please provide any specific examples or experiences you have. |
|  | Could you share the kinds of challenges you've noticed patients facing while accessing and navigating access to the health system? | Are there any adjustments that the practice or the hospital has put in place or need to be put in place to address these challenges? |
| **Deprivation and rurality** | How do you think deprivation impacts, if at all, the diagnosis and management of kidney disease in your patients? | How does this context influence your approach to care or the strategies you employ? |
|  | Can you share any observations or experiences regarding the management of kidney disease among populations in more deprived areas? | Are there specific challenges or adaptations for people you've noticed in your practice in managing kidney disease in more deprived areas? |
| **Adjustments and achieving equitable care** | What adjustments, if any, have you made at your practice/ cluster (area), with your board to provide fair and high-quality care to all kidney disease patients? | Are there things in your practice you would like to or could do differently when you recognise monitor and coordinate the care of people with kidney disease who are from poor backgrounds or have accessibility barriers (e.g., hearing, sight, mobility, transport, language, culture). Would that kind of adaption be possible? |
|  | What have you found needs to be in place to ensure help is provided timely and equitable care for people across your patient population with kidney disease? | - Can you describe any initiatives, resources, or strategies your practice has employed or found useful in ensuring equitable care for all patients, especially considering diverse health and social needs?" - In day-to-day practice, how is social support integrated, and what does this entail in terms of meeting individual needs and promoting equity? - Could you provide examples of any adjustments or trade-offs you've had to navigate to promote equity, whether during consultations, within the practice, or in the wider community context? |
| **Data and quality improvement** | Can you discuss any "learning opportunities" or training sessions your team has engaged in related to kidney disease? | Could you describe the scope of that training in terms of considering and addressing the diverse personal living circumstances of patients? |
|  | How would you know whether you were providing good care to all, or only to some and were leaving other people behind?  Are there areas in kidney disease care where you think more data would be beneficial? | - Are there aspects of the organisation of your team that particularly cater to or possibly overlook populations with certain backgrounds? - How do you use data for quality improvement in kidney disease management? |
